# Direct indicators of social distancing effectiveness in COVID-19 outbreak stages: a correlational analysis of case contacts and population mobility in Korea

**DOI:** 10.4178/epih.e2023065

**Published:** 2023-07-10

**Authors:** Sojin Choi, Chanhee Kim, Kun-Hee Park, Jong-Hun Kim

**Affiliations:** 1 Gyeonggi Infectious Disease Control Center, Health Bureau, Gyeonggi Provincial Government, Suwon, Korea; 2 Department of Social and Preventive Medicine, Sungkyunkwan University School of Medicine, Suwon, Korea

**Keywords:** COVID-19, Close-contact, Social distancing, Stringency, Indicators

## Abstract

**OBJECTIVES:**

The effectiveness of social distancing during the coronavirus disease 2019 (COVID-19) pandemic has been evaluated using the magnitude of changes in population mobility. This study aimed to investigate a direct indicator—namely, the number of close contacts per patient with confirmed COVID-19.

**METHODS:**

From week 7, 2020 to week 43, 2021, population movement changes were calculated from the data of two Korean telecommunication companies and Google in accordance with social distancing stringency levels. Data on confirmed cases and their close contacts among residents of Gyeonggi Province, Korea were combined at each stage. Pearson correlation analysis was conducted to compare the movement data with the change in the number of contacts for each confirmed case calculated by stratification according to age group. The reference value of the population movement data was set using the value before mid-February 2020, considering each data’s characteristics.

**RESULTS:**

In the age group of 18 or younger, the number of close contacts per confirmed case decreased or increased when the stringency level was strengthened or relaxed, respectively. In adults, the correlation was relatively low, with no correlation between the change in the number of close contacts per confirmed case and the change in population movement after the commencement of vaccination for adults.

**CONCLUSIONS:**

The effectiveness of governmental social distancing policies against COVID-19 can be evaluated using the number of close contacts per confirmed case as a direct indicator, especially for each age group. Such an analysis can facilitate policy changes for specific groups.

## GRAPHICAL ABSTRACT

[Fig f7-epih-45-e2023065]

## INTRODUCTION

An unprecedented catastrophic pandemic has raged worldwide since the first coronavirus disease 2019 (COVID-19) case was reported in Wuhan, China, on December 31, 2019 [1]. Since the first COVID-19 case was confirmed on January 20, 2020, in Korea, the cumulative number of confirmed cases had reached 16,929,564 by April 25, 2022, with most of these cases registered in metropolitan areas; furthermore, the 0.13% case-fatality rate necessitated the mandating of various policies as Korea’s COVID-19 response strategy, which was mainly based on case occurrences in metropolitan areas, to prevent the spread of severe acute respiratory syndrome coronavirus 2 (SARS-CoV-2) [2,3]. Gyeonggi Province, which surrounds Seoul, is the most populous administrative district (26.3% of the national population) in Korea [4]. Reducing social contact prevents the spread of infection, and government interventions decrease the effective reproductive number [5-7]. Social distancing, a key non-pharmaceutical intervention, helps decrease contact with carriers and is an essential public health and social measure in Korea [8]. Credit card usage and population movements from mobile geolocation data have been analyzed as indirect indicators of close contact to assess the impact of social distancing on SARS-CoV-2 spread [9,10]. Furthermore, the assessment of mobility patterns demonstrates the effectiveness of social distancing, and the transmission risk of COVID-19 can be assessed using data on average travel distance and residence time [11,12]. However, these approaches have a limited ability in allow inferences on individual behavior, including close interpersonal contact, because they lack demographic information of the patients with confirmed disease status [13,14]. As the Korean government has continuously implemented contact tracing to prevent infection spread, information on confirmed cases and close contacts has been accumulated and can be linked.

In this study, we examined the changes in the number of contacts per confirmed case to determine whether this information can be used as a direct indicator of the effectiveness of social distancing policies rather than the currently available indirect indicators, such as credit card usage and population movements from mobile geolocation.

## MATERIALS AND METHODS

### Data source

Data on confirmed cases and close contacts from the local public health center collected by the center in-charge and epidemiological investigation officers (EIOs) in Gyeonggi Province were analyzed. Population movement changes in response to social distancing in the metropolitan areas in Korea from February 15, 2020 to October 31, 2021, were assessed using data from four mobility datasets, from two Korean mobile communication companies, SK Telecom and KT Corporation (corporate mobile data were obtained from the Korea Statistical Data Center [data.kostat.go.kr] and Seoul Open Data Plaza [data.seoul.go.kr], respectively) and Google Community Mobility Reports (https://www.google.com/covid19/mobility/).

### Case definition

According to the Korea Disease Control and Prevention Agency guidelines, a confirmed COVID-19 case was defined as the detection of SARS-CoV-2 using real-time reverse transcription-polymerase chain reaction, regardless of clinical characteristics [15]. Close contacts included those in actual or suspected close contact with patients with confirmed COVID-19. During the epidemiological investigation of patients with confirmed COVID-19, the EIOs evaluated the environmental circumstances at the time of contact to determine whether close contact with others had occurred [15].

### Movement data

The baseline activity for 2 weeks (February 1-14, 2020) was collected to determine the percentage change in the proportions of visitors from other cities to Gyeonggi Province using SK Telecom mobility data, whereas the rate of change in the proportions of visitors to Seoul from Gyeonggi Province was determined using KT Corporation mobility data. Using Google’s Community Mobility Reports for Korea, data on changes in mobility trends from February 15, 2020 to October 31, 2021 were obtained by comparing the average baseline activity for the corresponding day of the week from January 3, 2020 to February 6, 2020, as the percentage change in visits to or time spent in: (1) transit places (i.e., public transportation hubs, including subway, bus, and train stations) in metropolitan areas and (2) multi-purpose facilities (e.g., restaurants, cafes, shopping centers, amusement parks, museums, and libraries).

### Social distancing policy data

Social distancing was implemented as a policy to help control the spread of the pandemic by limiting close contact among individuals, in response to the increasing number of confirmed COVID-19 cases in Korea. Table 1 shows the stages and main characteristics of social distancing from week 7 in 2020 to week 43 in 2021 in metropolitan areas [16].

### Statistical analysis

From the data collected for the residents of Gyeonggi Province, the numbers of confirmed cases and close contacts were stratified by weeks and linked with the weekly percentage change of movement using mobility data. Individuals were divided into nine age groups (< 6, 7-12, 13-18, 19-29, 30-39, 40-49, 50-59, 60-69, and ≥ 70 years) according to the period corresponding to the social distancing stage. According to the 12-stage social distancing policy from March 22, 2020 to October 31, 2021, the number of close contacts per confirmed case by age group was calculated. Using four mobility data sources (SK Telecom movement, KT Corporation movement, Google Transit, and Google Retail and Recreation), movement percentage changes were calculated by the 12 stages. The change in the amount of movement in each data source was calculated based on the baseline value in each data source. Pearson correlation analysis was conducted to compare the value of movement percentage changes with the change in the number of contacts for each confirmed case calculated by stratification according to age group. Statistical analysis was conducted using SAS version 9.4 (SAS Institute Inc., Cary, NC, USA), and R version 4.1.0 (R Foundation for Statistical Computing, Vienna, Austria) was used for visualization.

### Ethics statement

This study used quarantine data acquired through legally mandated public health investigation authorized under the Information Disease Control and Prevention Act (Nos. 12444 and 13392). This research protocol was approved by the Institutional Review Board of Sungkyunkwan University School of Medicine (IRB No. 2022-02-029). The informed consent was waived because all data were obtained as a result of a public health response.

## RESULTS

### Social distancing stages

Movement restrictions in Gyeonggi Province commenced on March 22, 2020, and social distancing included the reinforcement of prevention in daily life. From August 16, 2020, 3-step, 5-step, and 4-step systems were introduced according to the number of confirmed cases (Table 1). Information on variants of concern, simplified descriptions of changes in the social distancing policy, and the number of cases of COVID-19 are attached as an appendix (Supplementary Material 1).

### Changes in the number of confirmed cases and population movement according to the social distancing policy

**Figure 1.**
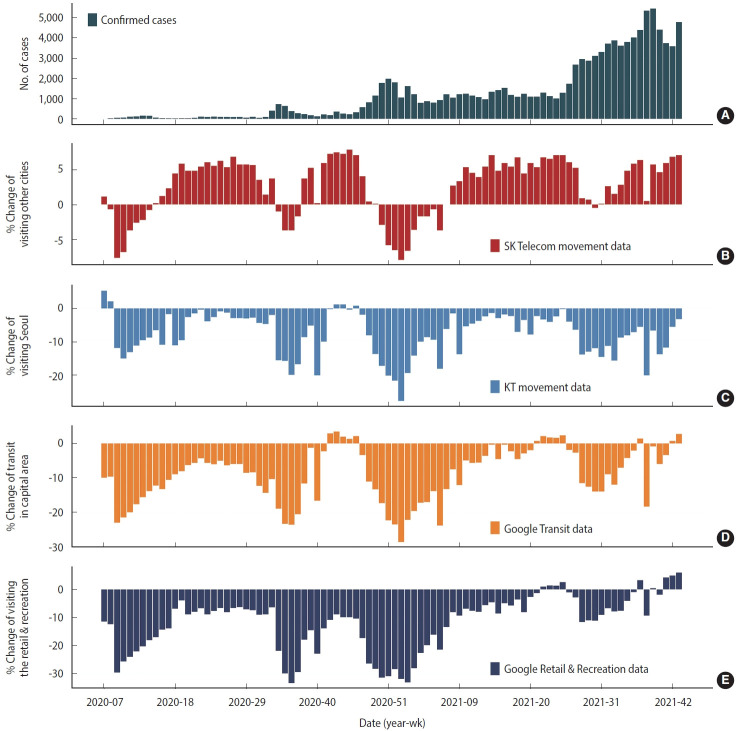
Changes in the number of coronavirus disease 2019 confirmed cases (A) and population movement (B: SK Telecom movement, C: KT movement, D: Google Transit, and E: Google Retail & Recreation) in Gyeonggi Province from week 7 of 2020 to week 43 of 2021.

The number of confirmed cases increased from 1 to 157 (weeks 7-12 in 2020) before systematic social distancing was implemented, and population movement changes of -2.6% and -11.2% (SK Telecom and KT data, respectively) compared to baseline were observed (Figure 1); Google Transit and Google Retail and Recreation data showed that population movement decreased by -17.7% and -22.1%, respectively. During stage 1 social distancing, the weekly number of confirmed COVID-19 cases decreased from 157 to 13, with associated increments in population movement (SK Telecom data: from -2.2 to +4.4%; Google Transit data: from -15.7 to -9%; and Google Retail and Recreation data: from -20.3 to -6.9%). In contrast, the KT data revealed that population movement decreased from -9.6% to -11.1%. During stages 3 to 4, the number of confirmed COVID-19 cases increased from 90 to 727 from week 32, 2020 to 34, 2020, resulting in an outbreak in Gyeonggi Province, where schools were partially closed and social distancing was reinforced (e.g., online classes); population movement decreased sharply (SK Telecom data: from +3.7 to -1.7%; KT data: from -2.0 to -16.8%; Google Transit data: from -10.4 to -20.6%; and Google Retail and Recreation data: from -6.4 to -29.4%). During stages 5-7, the weekly average number of confirmed COVID-19 cases was 270, and the social distancing stage was gradually relaxed from step 2.5 to step 1, and population movement increased (SK Telecom data: from +3.7 to +4.0%; KT data: from -8.7 to -1.9%; Google Transit data: from -11.7 to -3.4%; and Google Retail and Recreation data: from -17.9 to -17.4%). However, the number of COVID-19 cases surged from week 47 in 2020, and social distancing was reinforced until week 6 in 2021, corresponding to stages 8-9, and population movement decreased accordingly (SK Telecom data: from +4.0 to -3.7%; KT data: from -1.9 to -18.2%; Google Transit data: from -3.4 to -23.9%; and Google Retail and Recreation data: from -17.4 to -21.4%). For 5 months (weeks 7-27, 2021), the number of confirmed COVID-19 cases stabilized at approximately 1,200; social distancing (corresponding to stage 10) was relaxed by 1 step compared to the previous stage, but population movement in the KT data showed a minimal change (KT data: from -6.2 to -6.5%), whereas the movements in the other data showed an increase (SK Telecom data: from 0.0 to +5.2%; Google Transit data: from -13.3 to -2.7%; and Google Retail and Recreation data: from -13.4 to -2.9%). Since week 27 in 2021, corresponding to stages 11 and 12, the number of confirmed COVID-19 cases exceeded 2,000, and thus the social distancing policy was strengthened to step 4 (2 steps higher than in the previous stage). Population movement increased overall despite the strengthening of social distancing (SK Telecom data: from +0.9 to +7.0%; KT data: from -6.5 to -3.2%; Google Transit data: from -2.7 to +2.7%; and Google Retail and Recreation data: from -11.6 to +6.0%).

### Close contacts per confirmed case by social distancing stage stratified by age group

**Figure 2.**
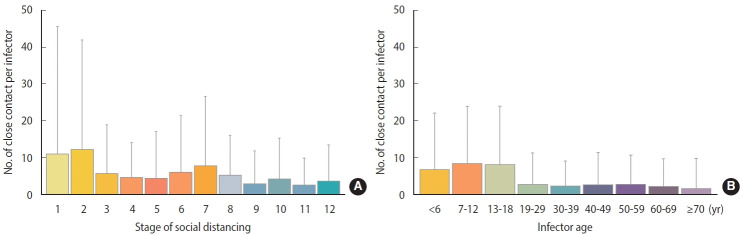
Number of close contacts per confirmed case (A) by social distancing stage and (B) infected person’s age group.

**Figure 3.**
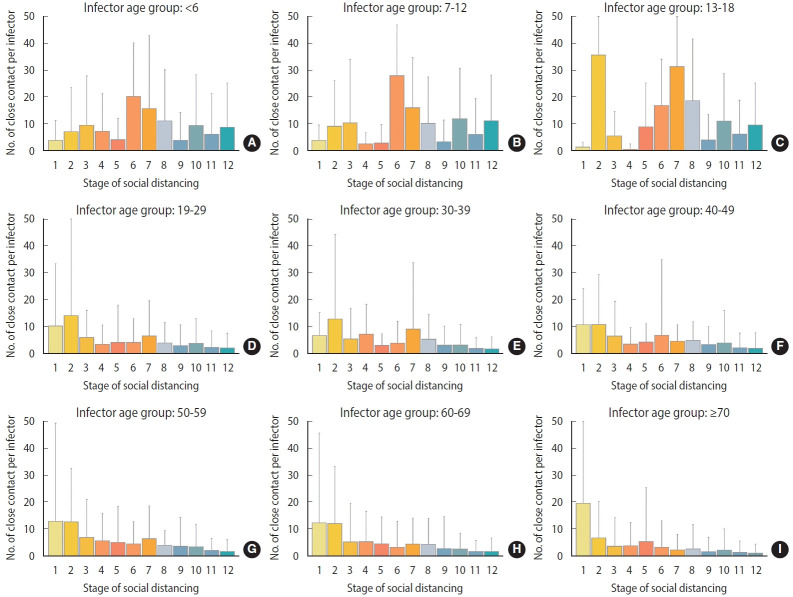
Number of close contacts per confirmed case according to each age group (A: <6, B: 7-12, C: 13-18, D: 19-29, E: 30-39, F: 40-49, G: 50-59, H: 60-69, and I: ≥70 years) and social distancing stage change.

The number of close contacts per confirmed case at each social distancing stage changed depending on whether social distancing was strengthened or relaxed. In stages 1 to 2, wherein social distancing was relaxed, the number of close contacts per confirmed case increased (11.08± 34.46→12.28± 29.57; Figure 2A), whereas stages 2 to 4 involved strengthening of social distancing, and the number of close contacts per confirmed case decreased (12.28± 29.57→4.72± 9.45). Stages 4 to 7 involved relaxation of social distancing, and the number of close contacts per confirmed case increased (4.72± 9.45→7.92± 18.68). Stages 7-9 involved strengthening social distancing, and the number of close contacts per confirmed case decreased (7.92± 18.68→3.02± 8.87). Stages 9-12 sequentially involved relaxed, strengthened, and relaxed social distancing, wherein the number of close contacts per infected person increased (3.02± 8.87→4.30± 11.10), decreased (4.30± 11.10→ 2.66± 7.33), and increased (2.66± 7.33→3.73± 9.85) sequentially. During the study period, the number of close contacts per confirmed case was two to three times higher in the 18 and under age group than in the 19 and over age group (0-6: 6.94± 15.16; 7-12: 8.60± 15.35; 13-18: 8.28± 15.71; 19-29: 2.85± 8.56; 30-39: 2.43±6.84; 40-49: 2.79 ± 8.76; 50-59: 2.89 ± 7.94; 60-69: 2.33 ± 7.55; ≥ 70: 1.77± 8.20; Figure 2B). The average change in the number of close contacts per confirmed case in each stage of social distancing was 8.5 in the age group of 18 years or younger, which was 4.5 times higher than that in the above-19 age group (1.9) (Figure 3).

### Correlation of changes in the number of close contacts per confirmed case according to 12 stages of social distancing for all two selectable age groups

**Figure 4.**
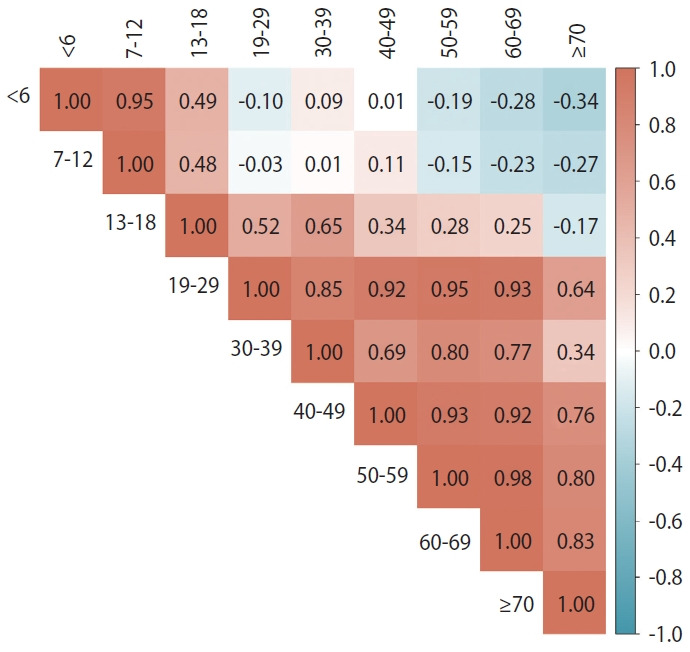
Correlation matrix for each age group against the changes in the number of close contacts per confirmed case according to the social distancing stage.

Figure 4 shows the correlations of the changes in the number of close contacts per confirmed case according to the social distancing stage between different age groups. Among those 18 years of age or younger, the 0-6-year-old and 7-12-year-old groups were positively correlated (r= 0.95), the 0-6-year-old and 13-18-year-old groups were positively correlated (r= 0.49), and the 7-12-year-old and 13-18-year-old groups were positively correlated (r= 0.48). However, two age groups of 12 years of age or younger and each age group of ≥ 19-year-olds had more negative correlations overall. Among the 13 and older age groups, most of the two age group comparisons showed positive correlations.

### Correlation between changes in population movement and changes in the number of close contacts per confirmed case by each age group, depending on whether or not data after the vaccination period were included

**Figure 5.**
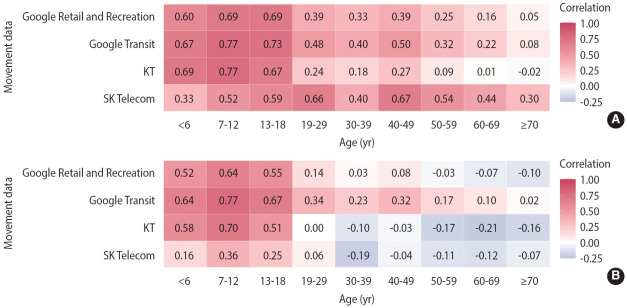
Correlation between each movement data and the number of close contacts per confirmed case by age group. (A) Correlation from stage 1 to stage 9 (before the vaccination period in the adult’s age group), (B) correlation from stage 1 to stage 12 (including the vaccination period in the adult’s age group).

The correlation between the number of close contacts per confirmed case and the population movement according to the changes in the social distancing stage changed after stage 9 (Figure 5). In each group of those 18 years of age or younger, the correlations between the number of close contacts per confirmed case and the population movement in Google Retail and Recreation data (0-6 years: 0.60; 7-12 years: 0.69; 13-18 years: 0.69), Google Transit data (0.67; 0.77; 0.73), KT data (0.69; 0.77; 0.67), and SK Telecom data (0.33; 0.52; 0.59) remained high during stages 1-9 (Figure 5A). In contrast, during stages 1-12, the correlations between the number of close contacts per confirmed case and the population movement in Google Retail and Recreation data (0.52; 0.64; 0.55), Google Transit data (0.64; 0.77; 0.67), KT data (0.58; 0.70; 0.51), and SK Telecom data (0.16; 0.36; 0.25) showed a slight decrease, although a relatively high correlation was maintained (Figure 5B). On the other hand, in each age group of ≥ 19-year-olds, a weak positive correlation, from 0.01 to 0.67, was noted, except for the KT data (-0.02 for > 70 years) during stages 1-9 (Figure 5A). In stages 1-12 including the stages 10-12 period, in each age group of ≥ 19-year-olds, the positive correlation was a minimum of 0.02 (Google Transit data for ≥ 70-year-old group) and a maximum of 0.34 (Google Transit data, 19-29-year-old group). However, when compared with the results including only stages 1-9 (Figure 5A), the positive coefficient values disappeared, and negative correlation values were shown in over half of the total: from a minimum of -0.21 (KT data of the 60-69-year-old group) to a maximum -0.03 (Google Retail and Recreation data of the 50-59-year-old group; Figure 5B).

### Changes in the number of close contacts per confirmed case in those 18 years of age or younger and changes in population movement, according to social distancing policy

**Figure 6.**
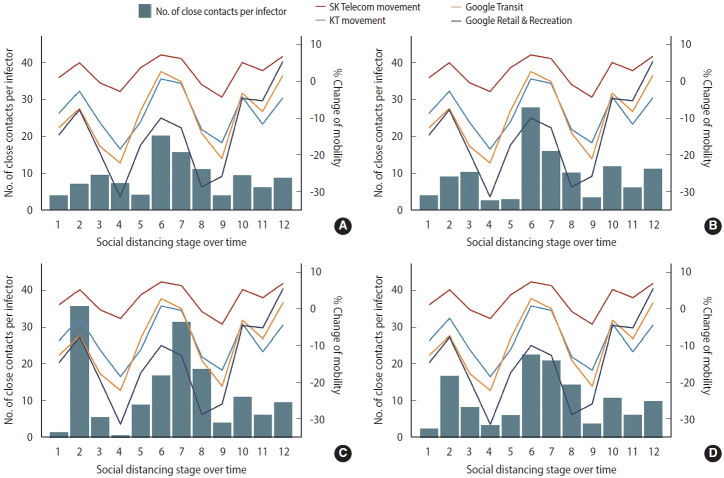
Change in social distancing stages over time, amount of movement each stage entails, and number of close contacts per confirmed case by age group. (A) 0-6 years old, (B) 7-12 years old, (C) 13-18 years old, (D) 0-18 years old.

In each age group of those 18 years of age or younger, the changes in population movement according to the social distancing stage and the changes in the number of close contacts per confirmed case were similar among the different age groups (Figure 6). The entire group of those 18 years of age or younger and the two subgroups therein had the highest numbers of close contacts per confirmed case in stage 6 (≤ 18 years: 22.65± 19.15; 0-6 years: 20.33±19.88; 7-12 years: 28± 18.86; Supplementary Material 2). The % change of mobility data of SK Telecom (+7.27%), KT (+0.73%), and Google Transit (+2.73%) also showed the highest values in same stage 6 (Supplementary Material 3).

## DISCUSSION

This study confirmed that the trend of the number of close contacts per confirmed case in Gyeonggi Province was similar to changes in the actual movement of Gyeonggi Province residents. The study also found that this trend varied according to the stage of social distancing policy applied in the Seoul metropolitan area. The trend in the number of close contacts is an important achievement of the effectiveness of social distancing, as demonstrated by the investigation of confirmed case numbers and their impact on infection prevention. Understanding this trend can help inform social distancing policies and prevent the spread of infectious diseases [17-19]. The social distancing policy in Korea restricted citizens from visiting crowded places and from coming into close contact with others [20,21]. Many other countries implemented social distancing policies during the COVID-19 pandemic, but the stringency of their measures varied depending on the number of confirmed cases and their unique community structures, economies, cultures, and health systems. Some countries enforced strict policies, while others were more lenient. Despite the severity of COVID-19 being similar across the world, each country faced different challenges and made decisions accordingly [19,22].

From week 7 in 2020 to week 43 in 2021, while the number of confirmed COVID-19 cases increased and decreased, the population movement as per the four mobility data sources (SK Telecom, KT, Google Transit, and Google Retail and Recreation) changed similarly. This proves that actual population movement was correlated with the spread of SARS-CoV-2 [23]. If a social distancing policy is implemented at an appropriate time, the population movement rate decreases, and the number of confirmed cases decreases [24-27]. However, as the COVID-19 pandemic continued for a long time, the movement of the population gradually changed. From late 2020 to mid-2021, as the number of cases remained stable without significant fluctuations, and the population’s mobility gradually increased. In early stage 11, as the number of confirmed cases abruptly increased, the level of population movement rapidly decreased. However, from week 33, 2021, during mid-stage 11, despite an increase in the number of confirmed cases, there was a corresponding increase in population movement, suggesting that the impact of social distancing policies had gradually disappeared. This trend is consistent with the public’s growing fatigue of social distancing and decreasing awareness of COVID-19 [28-30].

The number of close contacts per confirmed case differed for the 12 stages, depending on the age of the infected person. In the early stages, the number of close contacts per confirmed case in the above-19 age group initially remained high and subsequently decreased in the second half of the 12 stages, as vaccination of those aged ≥ 19 years commenced after stage 9. This is because, fully vaccinated individuals were excluded from the number of close contacts, even if they were themselves close contact with the infected person, owing to the presumed effectiveness of the vaccine in preventing the spread of the infection [31,32].

The number of people coming into contact with confirmed COVID-19 cases in each age group changed differently during each stage of social distancing. The rate of change in the age group of those 18 years of age or younger was higher than that of the over-19 years of age in accordance with the social distancing stage. This is due to the school closure as measures of social distancing that suppressed close contact between students at school, which plays a decisive role in the spread of infection within the peer group. This is consistent with previous data that revealed a difference in the close contact rate depending on school closure: closing entire grades was more effective in preventing spread than closing only a few classes [33].

Behavioral changes in response to social distancing policies differed between adults (≥ 19 years) and younger/pediatric groups (≤ 18 years). Children and teenagers spend a considerable amount of time in groups at childcare facilities and schools, which may have affected their behavior differently than that of adults. Studies have shown that people of the same age group tend to have more contact with individuals of similar age during the day [34,35]. Policies that limit the number of people in restaurants or gatherings may have less impact on behavior than policies that affect the proportion of students attending school. Furthermore, research suggests that adults aged 19 and older tend to have shorter periods of contact with others than children and teenagers, who spend more time in communal settings like schools and childcare facilities [36]. Therefore, for the younger/pediatric group (≤ 18 years), policy effectiveness can be observed mainly in the public domain, whereas for the adult group, policy effectiveness is greater in the private domain. Moreover, unlike students, no strong restrictions were placed on economically active people who were working.

Changes in national policies affected the number of close contacts per confirmed case. As mass vaccination commenced around stage 10 of social distancing in Korea (February 26, 2021), the policy was changed such that close contacts who were fully vaccinated were excluded from the number of close contacts, even if they were in close contact with confirmed case after May 5, 2021. According to the first edition of the Korean government’s guidelines for vaccine completion, self-quarantine was not applicable for those who were fully vaccinated against COVID-19 [37].

For those 18 years of age or younger, the sensitivity to the change in the number of close contacts per confirmed case for each stage 1-12 was high because most of them had not received a COVID-19 vaccine by the time of stage 12 and due to the relatively minor effect of the COVID-19 vaccination program, compared to that in other age groups [38]. Therefore, the number of close contacts per confirmed case in these age groups displayed a high correlation with population movement data. The rate of close contact correlates directly with the likelihood of disease transmission through direct contact or respiratory droplets; therefore, identifying close contact is more beneficial than ascertaining mobility indicators [39]. The number of close contacts per confirmed case was a better indicator than the amount of population movement. Thus, the number of close contacts for each confirmed case can be a more useful tool for assessing the effectiveness of social distancing policies than population movement.

This study had the following limitations. First, as the population movement data were not stratified by age group, a direct comparison of the correlation with the number of close contacts per confirmed case by age group was not possible. Second, owing to changes in the close contact policy of social distancing, fully vaccinated individuals were exempt from self-quarantine after May 5, 2021. Third, the criteria for close contact can be applied in various ways according to the subjective judgment of EIOs, and there is a possibility that less stringent criteria were applied in the late stage than in the early stage. Fourth, it is likely that it was much easier to identify close contacts for the younger/pediatric group (≤18 years), who spent a considerable amount of their time in educational facilities, such as schools and childcare facilities, than for the adult group. This may be related to the fact that a large number of close contacts was found per confirmed case in the younger/pediatric group.

Despite these limitations, we confirmed that the effects of social distancing policy might differ among age groups, regardless of population movement. The effect of restrictions on school attendance was more pronounced than that of restrictions on restaurants and gatherings. We compared the number of close contacts per confirmed case with the data on population movement and found that changes in the number of close contacts per confirmed case can be used as a valuable index to evaluate the effectiveness of the social distancing policy.

In this study, we examined the possibility of using the number of close contacts per confirmed case as a direct indicator to evaluate the effectiveness of social distancing. Close contacts of each confirmed case are investigated with the goal of finding those who should be quarantined. Therefore, to use this indicator to monitor the effectiveness of social distancing, complementary measures will be needed with consideration of the factors that cause differences by age group in this study. More specifically, even if quarantine is not necessary because people have been vaccinated, those people should be included in the list of close contacts anyway. Changes in the number of contacts per confirmed case can serve as an excellent direct indicator to evaluate the effectiveness of social distancing in a situation where epidemiological investigations are systematically conducted, and close contacts of confirmed cases are thoroughly investigated.

## Figures and Tables

**Figure f7-epih-45-e2023065:**
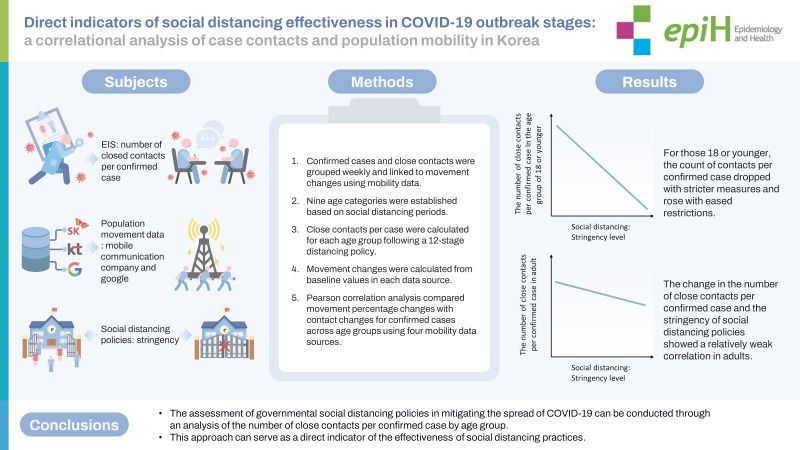


**Table 1.** Social distancing system in Gyeonggi Province for COVID-19 control from week 7 in 2020 to week 43 in 2021

Period (stage)	Social distancing system		Main features
Mar 22, 2020 to May 5, 2020 (stage 1)	Enhanced social distancing		- Refrain from going abroad, minimization of crowded environments such as telecommuting for business, and recommendation to suspend operations in religious facilities, indoor sports facilities, and nightlife facilities, etc.
			- Strengthen quarantine management for high-risk groups such as nursing hospitals, psychiatric hospitals, and churches
			- Refrain from unnecessary gatherings, going out, and events as much as possible, but if unavoidable, allow limited access to the extent of observing quarantine guidelines
			- Existing administrative orders for religious facilities, some sports facilities, nightlife facilities, and private institutes are maintained, but adjustments are made with recommendations to refrain from operation, and orders to comply with quarantine guidelines are maintained
May 6, 2020 to Aug 15, 2020 (stage 2)	Prevention in daily life		- Conduct gatherings, meetings, and events in compliance with quarantine rules
			- Multi-use facilities to comply with the quarantine rules and are operated or partially restricted
			※ However, for high-risk facilities, an administrative order to comply with quarantine rules, such as preparation of an electronic access list, etc.
Aug 16, 2020 to Aug 30, 2022 (stage 3)	The three-step system (1, 2, 3)	Step 2	- Gatherings, meetings, and events of more than 50 people indoors and more than 100 people outdoors are prohibited, and sports events cannot have spectators
			- 12 types of high-risk facilities such as clubs, karaoke, and buffets prohibited from allowing gatherings
			- Suspension of indoor public facilities operations
			- Metropolitan churches to conduct non-face-to-face worship
Aug 31, 2022 to Sep 13, 2020 (stage 4)		Step 2.5	- Only take-out and delivery are allowed between 21:00 and 5:00 the next day from restaurants and bakeries
			- Prohibition of gatherings at private institutes, reading rooms, study cafes, vocational training institutions, and indoor sports facilities
Sep 14, 2020 to Oct 11, 2020 (stage 5)		Step 2	- Gatherings, meetings, and events of more than 50 people indoors and 100 people outdoors are prohibited
			- For multi-use facilities such as PC rooms, core quarantine rules such as management of the visitor list are mandatory
			- In the metropolitan area, existing measures such as the ban on gatherings at high-risk facilities such as clubs, bars and door-to-door sales continue
			- In the metropolitan area, small gatherings and meals at churches are still prohibited, and non-face-to-face worship required
Oct 12, 2020 to Nov 6, 2020 (stage 6)		Step 1	- 50 people indoors and 100 people outdoors; limit 1 person per 4 m^2^ of facility area
			- Mandatory 11 quarantine rules and 16 quarantine rules for restaurants and cafes
			- Face-to-face worship is possible, but the number of people is limited, gatherings and meals are prohibited
			- Limited number of spectators at sporting events
Nov 7, 2020 to Nov 23, 2020 (stage 7)	The five-step system (1, 1.5, 2, 2.5, 3)	New step 1	- Reinforcement of mandatory rules for key management facilities and general management facilities (limit of 1 person per 4 m^2^ of facility area, etc.)
			- Gatherings and events with more than 500 people must be reported to local authorities; mandatory quarantine rules with ban on gatherings, festivals, large-scale concerts, and academic events with more than 100 people
			- Worship limited to 30% of the number of seats; no gatherings and meals allowed
Nov 24, 2020 to Dec 7, 2020 (stage 8)		New step 2	- Suspension of operation after 21:00, such as indoor sports facilities and karaoke
			- Cafes to allow only take-out and delivery; restaurants to allow only take-out and delivery after 21:00, etc.
			- Number of people limited to less than 100 per individual at wedding/funeral ceremony, etc.
Dec 8, 2020 to Feb 14, 2021 (stage 9)		New step 2.5	- Ban on gathering at 6 types of nightlife facilities
			- Less than 50 people per event
			- Limited to 10% or less seats for regular worship service
Feb 15, 2021 to Jul 11, 2021 (stage 10)	The four-step system (1, 2, 3, 4)	New step 2	- Private gatherings of up to 4 people allowed; public gatherings and events of up to 99 people allowed
			- Ban on gatherings at nightlife facilities; cessation of operations after 22:00, such as karaoke bar
			- Only take-out and delivery allowed after 22:00 from restaurants and cafes
Jul 12, 2021 to Oct 17, 2021 (stage 11)		New step 4	- Private meetings can be held for up to 4 people who have not been vaccinated and up to 8 people including those who have been vaccinated without any time restrictions
			- Restrictions on 24-hours operation, such as reading rooms and performance halls
			- Up to 250 people (49 people + 201 people who have completed vaccinations) regardless of whether a wedding meal is provided
			- If meals are not provided, a total of 199 people can be present (99 people+100 people who have completed vaccination)
			- Up to 10% of the total capacity of religious facilities; up to 20% for only those who have completed vaccination
Oct 18, 2021 to Oct 31, 2021 (stage 12)		Step 4 towards a return to normal life	- Private meetings can be held for up to 4 people who have not been vaccinated and up to 8 people including those who have been vaccinated without any time restrictions
			- Restrictions on 24-hour operations, such as reading rooms and performance halls
			- Up to 250 people (49 people + 201 people who have completed vaccination) regardless of whether a wedding meal is provided
			- A total of 199 people can be accommodated if no meals are provided (99 people+100 people who completed vaccination)
			- Up to 10% of the total capacity of religious facilities; up to 20% for only those who have completed vaccination
