# Supplementary material for: Direct indicators of social distancing effectiveness in COVID-19 outbreak stages: a correlational analysis of case contacts and population mobility in Korea
Source: Epidemiol Health. 2023 Jul 10;45:e2023065. doi: 10.4178/epih.e2023065 (PMC10876423; doi:10.4178/epih.e2023065)
Supplement: Supplementary Material 1. [file epih-45-e2023065-Supplementary-1.docx]

Supplementary Table 1. (1) Mutation pattern in Korea^1^, (2) Change in social distancing policy in Gyeonggi Province, Korea, and (3) Number of cases of COVID-19 in Korea^2^

| Year and month | Variants of Concern (VOC)  No. of VOC (detection rate %) | | | | Social distancing system | Confirmed cases |
| --- | --- | --- | --- | --- | --- | --- |
|  | Alpha (GRY) | Beta (GH) | Gamma (GR) | Delta (GK) |  |  |
| 2020-02 | 0 | 0 | 0 | 0 | Enhanced social distancing | 2920 |
| 2020-03 | 0 | 0 | 0 | 0 | Enhanced social distancing (2020-03-22 to 2020-05-05) | 6855 |
| 2020-04 | 0 | 0 | 0 | 0 | Enhanced social distancing (2020-03-22 to 2020-05-05) | 979 |
| 2020-05 | 0 | 0 | 0 | 0 | Enhanced social distancing (2020-03-22 to 2020-05-05) | 703 |
| 2020-06 | 0 | 0 | 0 | 0 | Prevention in daily life (2020-05-06 to 2020-08-15) | 1331 |
| 2020-07 | 0 | 0 | 0 | 0 | Prevention in daily life (2020-05-06 to 2020-08-15) | 1506 |
| 2020-08 | 0 | 0 | 0 | 0 | Prevention in daily life (2020-05-06 to 2020-08-15), Step 2 (2020-08-16 to 2020-08-30), Step 2.5 (2020-08-31 to 2020-09-13) | 5641 |
| 2020-09 | 0 | 0 | 0 | 0 | Step 2.5 (2020-08-31 to 2020-09-13), Step 2 (2020-09-14 to 2020-10-11) | 3865 |
| 2020-10 | 0 | 0 | 0 | 0 | Step 2 (2020-09-14 to 2020-10-11), Step 1 (2020-10-12 to 2020-11-06) | 2700 |
| 2020-11 | 0 | 0 | 0 | 0 | Step 1 (2020-10-12 to 2020-11-06), New Step 1 (2020-11-7 to 2020-11-23), New Step 2 (2020-11-24 to 2020-12-07) | 7688 |
| 2020-12 | 14 (2.8%) | 1 (0.2%) | 0 | 0 | New Step 2 (2020-11-24 to 2020-12-07), New Step 2.5 (2020-12-08 to 2021-02-14) | 26527 |

Continued

Supplementary Table 1 continued

| Year and month | Variants of Concern (VOC)  No. of VOC (detection rate %) | | | | Social distancing system | Confirmed cases |
| --- | --- | --- | --- | --- | --- | --- |
|  | Alpha (GRY) | Beta (GH) | Gamma (GR) | Delta (GK) |  |  |
| 2021-01 | 51 (8.3%) | 10 (1.6%) | 1 (0.2%) | 0 | New Step 2.5 (2020-12-08 to 2021-02-14) | 17471 |
| 2021-02 | 122 (10.8%) | 12 (1.1%) | 0 | 0 | New Step 2.5 (2020-12-08 to 2021-02-14), New Step 2 (2021-02-15 to 2021-07-11) | 11467 |
| 2021-03 | 146 (6.2%) | 23 (1.0%) | 2 (0.1%) | 0 | New Step 2 (2021-02-15 to 2021-07-11) | 13414 |
| 2021-04 | 516 (16.2%) | 71 (2.2%) | 1 (0.0%) | 46 (1.4%) | New Step 2 (2021-02-15 to 2021-07-11) | 18927 |
| 2021-05 | 804 (27.7%) | 23 (0.8%) | 2 (0.1%) | 122 (4.2%) | New Step 2 (2021-02-15 to 2021-07-11) | 18330 |
| 2021-06 | 792 (23.2%) | 3 (0.1%) | 9 (0.3%) | 639 (18.7%) | New Step 2 (2021-02-15 to 2021-07-11) | 16623 |
| 2021-07 | 600 (7.9%) | 5 (0.1%) | 5 (0.1%) | 4,105 (53.7%) | New Step 2 (2021-02-15 to 2021-07-11), New Step 4 (2021-07-12 to 2021-10-17) | 41374 |
| 2021-08 | 174 (1.3%) | 0 | 0 | 11,064 (85.7%) | New Step 4 (2021-07-12 to 2021-10-17) | 53076 |
| 2021-09 | 1 9 (0.1%) | 0 | 2 (0.1%) | 15,049 (98.5%) | New Step 4 (2021-07-12 to 2021-10-17) | 59856 |
| 2021-10 | 0 | 0 | 0 | 12,744 (99.9%) | New Step 4 (2021-07-12 to 2021-10-17), Step 4 as stepping toward (2021-10-18 to 2021-10-31) | 53413 |

^1^ COVID-19 [Press release]. Korean Ministry of Health and Welfare 2020. [cited 2022 Dec 30]. Available from: <https://www.kdca.go.kr/board/board.es?mid=a20501010000&bid=0015>

^2^ COVID-19. [Internet] Korean Ministry of Health and Welfare 2020. [cited 2022 Dec 30]. Available from: <http://ncov.kdca.go.kr>
