# Supplementary material for: Direct indicators of social distancing effectiveness in COVID-19 outbreak stages: a correlational analysis of case contacts and population mobility in Korea
Source: Epidemiol Health. 2023 Jul 10;45:e2023065. doi: 10.4178/epih.e2023065 (PMC10876423; doi:10.4178/epih.e2023065)
Supplement: Supplementary Material 2. [file epih-45-e2023065-Supplementary-2.docx]

Supplementary Table 2. Average number of contacts per confirmed patient aged 0–18 depending on social distancing in Gyeonggi Province, Korea

| Classification | | 0–18 years old | | Under 6 years of age | | 7–12 years old | | 13–18 years old | |
| --- | --- | --- | --- | --- | --- | --- | --- | --- | --- |
| Stage | Social distancing system | Mean | SD | Mean | SD | Mean | SD | Mean | SD |
| Stage 1 | Strengthening social distancing | 2.53 | 4.36 | 4.00 | 7.38 | 4.00 | 5.66 | 1.50 | 1.78 |
| Stage 2 | Prevention in daily life | 16.85 | 45.96 | 7.18 | 16.43 | 9.19 | 16.99 | 35.70 | 75.23 |
| Stage 3 | Step 2 | 8.38 | 17.73 | 9.59 | 18.37 | 10.45 | 23.62 | 5.60 | 9.18 |
| Stage 4 | Step 2.5 | 3.41 | 8.31 | 7.35 | 13.99 | 2.67 | 4.16 | 0.67 | 1.97 |
| Stage 5 | Step 2 | 6.14 | 12.50 | 4.23 | 7.81 | 3.00 | 6.88 | 9.00 | 16.30 |
| Stage 6 | Step 1 | 22.65 | 19.15 | 20.33 | 19.88 | 28.00 | 18.86 | 16.93 | 17.20 |
| Stage 7 | New Step 1 | 21.00 | 33.01 | 15.78 | 27.17 | 16.1 | 18.57 | 31.39 | 45.10 |
| Stage 8 | New Step 2 | 14.48 | 20.86 | 11.18 | 19.04 | 10.25 | 17.27 | 18.71 | 22.96 |
| Stage 9 | New step 2.5 | 3.87 | 9.38 | 3.99 | 10.37 | 3.45 | 8.06 | 4.10 | 9.56 |
| Stage 10 | New step 2 | 10.85 | 18.48 | 9.50 | 18.88 | 11.93 | 18.82 | 11.13 | 17.81 |
| Stage 11 | New step 4 | 6.25 | 13.58 | 6.27 | 15.06 | 6.20 | 13.26 | 6.28 | 12.59 |
| Stage 12 | Step 4 towards a return to normal life | 9.99 | 16.31 | 8.82 | 16.45 | 11.27 | 16.98 | 9.64 | 15.63 |
