# Supplementary material for: Direct indicators of social distancing effectiveness in COVID-19 outbreak stages: a correlational analysis of case contacts and population mobility in Korea
Source: Epidemiol Health. 2023 Jul 10;45:e2023065. doi: 10.4178/epih.e2023065 (PMC10876423; doi:10.4178/epih.e2023065)
Supplement: Supplementary Material 3. [file epih-45-e2023065-Supplementary-3.docx]

Supplementary Table 3. Mobility data from mobile service providers according to social distancing in Gyeonggi Province, Korea

| Classification | | SK Telecom | KT Corporation | Google transit | Google retail recreation |
| --- | --- | --- | --- | --- | --- |
| Stage | Social distancing system |  |  |  |  |
| Stage 1 | Strengthening social distancing | 1.04 | -8.7 | -12.60 | -14.60 |
| Stage 2 | Prevention in daily life | 5.13 | -2.61 | -7.35 | -7.70 |
| Stage 3 | Step 2 | -0.33 | -11.13 | -17.60 | -19.40 |
| Stage 4 | Step 2.5 | -2.70 | -18.4 | -22.15 | -31.35 |
| Stage 5 | Step 2 | 3.75 | -11.00 | -8.00 | -17.30 |
| Stage 6 | Step 1 | 7.27 | 0.73 | 2.73 | -9.90 |
| Stage 7 | New Step 1 | 6.27 | -0.50 | 0.00 | -12.60 |
| Stage 8 | New Step 2 | -0.80 | -13.03 | -13.97 | -28.70 |
| Stage 9 | New step 2.5 | -4.24 | -16.63 | -20.99 | -25.80 |
| Stage 10 | New step 2 | 5.19 | -4.16 | -3.16 | -4.60 |
| Stage 11 | New step 4 | 2.98 | -11.60 | -8.14 | -5.20 |
| Stage 12 | Step 4 towards a return to normal life | 6.90 | -4.40 | 1.70 | 5.50 |
